# Physical and mental health impacts of the COVID-19 pandemic among college students who are undocumented or have undocumented parents

**DOI:** 10.1186/s12889-021-11606-x

**Published:** 2021-08-21

**Authors:** Annie Ro, Victoria E. Rodriguez, Laura E. Enriquez

**Affiliations:** 1 Department of Health, Society, and Behavior, University of California, Irvine, CA USA; 2 Department of Chicano/Latino Studies, University of California, Irvine, CA USA

**Keywords:** COVID-19, Undocumented immigrants, Young adults, Mixed-status families

## Abstract

**Background:**

The COVID-19 pandemic may have disproportionately affected the mental and physical health of undocumented students and students with undocumented parents.

**Methods:**

We analyzed primary data from 2111 California college students collected March–June 2020. We estimated the odds of mental or physical health being affected “a great deal” by COVID by immigration group and then examined whether this was moderated by campus belonging or resource use.

**Results:**

Students with undocumented parents were least likely to report COVID-related mental and physical health effects. Undocumented students and students whose parents have lawful immigration status did not differ in their COVID-related physical and mental health. For all students, more campus resource use and higher campus belonging were associated with negative mental and physical health effects.

**Discussion:**

Negative COVID-related mental and physical health was widespread. Separation from campus-based resources was detrimental during the early stages of the pandemic.

**Supplementary Information:**

The online version contains supplementary material available at 10.1186/s12889-021-11606-x.

## Background

The COVID pandemic has completely reshaped higher education, with campus shut downs, shifts to remote learning, and curtailed campus life. These drastic and abrupt changes have created challenges that can threaten the mental health and well-being of college students [1]. Some empirical work has revealed that the COVID pandemic elicited substantial negative effects on college students’ health. In a large Texas survey, the vast majority of college students reported changes in sleeping habits and eating patterns due to the pandemic and nearly 80% reported any depression and 71% showed some anxiety [2]. Depression, anxiety, suicidal ideation, and substance use increased after the start of the pandemic among national samples of college students [3]. A qualitative study similarly found high levels depression and anxiety [4]. Students attributed their stress to concerns about their health, difficulty concentrating, and disruptions in sleeping patterns. On top of this, low-income, racial minority students report extraordinary stress from campus closures: loss of income from on-campus jobs, technology gaps, limited study space at home, increased family obligations, and psychological distress [5, 6].

The impact of COVID-related campus closures on mental health and well-being are likely to disproportionately affect students who are already at the margins, including those who are vulnerable due to their own or their parents’ liminal legal status. Undocumented students represent one out of every 50 students enrolled in postsecondary education in the United States; California hosts 20% of these college students [7]. There are no estimates of the number of U.S. citizen college students with undocumented parents, but this is likely a significant and growing student population. Estimates from 2013 suggest that 4.5 million U.S. citizen children have at least one undocumented parent [8]; this is the case for nearly one in eight K–12 school children in California [9].

Undocumented immigrants have limited access to healthcare and are overrepresented among essential workers [10]. Additionally, Latinos, who make up the majority of California’s undocumented population, have borne greater health and economic costs of COVID-19 [11, 12]. Further, federal relief funding has excluded undocumented immigrants and their citizen family members from receiving stimulus payments and barred undocumented college students from accessing emergency grant aid [13]. As with other collateral consequences of immigration policies, COVID-related vulnerabilities are shared with the U.S. citizen family members of undocumented immigrants [14–16].

The unequal burden of COVID-19 borne by undocumented students and families of undocumented immigrants comes amidst existing challenges in their pursuit of higher education. Higher education is a promising means to upward mobility for these students, but can also be a fraught time of heightened mental strain and poor physical health generally [17]. For undocumented students and U.S. citizen students with undocumented parents, these strains are magnified by the precarity of their or their family members’ legal status. Their families’ restricted economic and occupational mobility limits their financial support and students may even be expected to contribute financially to the household while they are in school [18, 19]. The increasingly restrictive immigration climate brings concerns for their own and their families’ safety from deportation. For undocumented students, these fears for their families may be more urgent than concerns for their own deportation [20]. Both groups may also develop feelings of stigma, shame, or guilt which, if internalized, can contribute to poorer mental health [21, 22].

One potential moderating factor in the mental and physical health impact of COVID-19 is the role of the campus environment. College campuses are not only sites where students learn, but where they live, work, and create relationships. There are two types of resources that campuses can provide. The first is psychological, with feelings of campus belonging, which encompasses students’ perceived level of social support, connectedness, and being important in their college community [23]. Campus belonging has been associated with better academic performance, persistence, and mental health among college students [23–25]. The second type is material, like academic support services or health care for students through on-campus clinics. Campuses are also increasingly providing resources to help meet students’ basic needs [26]. A recent study of more than 43,000 students found widespread basic needs insecurity among university students: 36% were food insecure, 36% were housing insecure, and 9% were homeless [27]. Campus shut down may have been especially hard for students who relied on campus resources for their needs; 60% of college students in a national survey reported that the pandemic made it more difficult to access mental health care [3]. Students who had high levels of campus belonging and were frequent users of campus resources prior to the pandemic may have experienced more severe COVID-related mental and physical health problems as campuses shut down. For undocumented students and citizen students with undocumented parents, losing access to these resources may have been even more detrimental, considering their heightened vulnerability to COVID-related stressors.

In this paper, we focus on how the COVID-19 pandemic affected the physical and mental well-being of college students with differing immigration-related vulnerabilities. We focus on self and parental immigration status as a central point of comparison because it is directly tied to an individual’s and family’s ability to cope with the pandemic. We have several hypotheses. First, we expect undocumented students and U.S. citizen students with undocumented parents to report poorer mental and physical health because of the COVID pandemic than their U.S. citizen peers whose parents have lawful immigration status (e.g. naturalized citizens, permanent residents). We further consider the moderating effects of campus resources, both psychological and material. We hypothesize that undocumented students and citizens with undocumented parents who have high levels of campus belonging and use more campus resources will show more negative COVID-related mental and physical health than citizens students with lawful status parents. Our results will help campuses identify which students are high risk for COVID-related physical and mental health issues and offer institutional support.

## Methods

This study uses survey data collected between March to June 2020 by the University of California Collaborative to Promote Immigrant and Student Equity (UC PromISE). Participants were children of immigrants who were undergraduate students attending the UC system. The UC system is composed of nine campuses spread out across the state, including urban, sub-urban, and rural campuses. It is the more selective of the two California university systems and hosts a more traditional college student population, but its campuses vary in selectivity and study body demographics. Participants were recruited through emails and social media posts from each campus’ undocumented student support services office, faculty teaching large general education courses and ethnic studies courses, departmental and university office newsletters, and undocumented student organizations. Eligibility criteria included being over age 18, having at least one immigrant parent, and current enrollment as a UC undergraduate student. The survey was administered via Qualtrics [28] with an estimated completion time of 25–35 min (see supplemental files for survey instrument). Respondents received compensation via a $10 electronic gift card.

There were a total 2769 total survey respondents, of which 2331 were asked the COVID questions. We used list wise deletion to preserve respondents with non-missing values for all of the variables described in the two multivariate models below. The levels of missing for each variable were below 5%; the variable with most missing was gender (2.1%). Our final analytic sample size was 2111 (667 undocumented immigrant students, 648 U.S. citizen students with at least one undocumented parent, and 1427 U.S. citizen students whose parents have lawful immigration status). All project activities were approved by the UC Irvine Institutional Review Board.

### Variables

#### Mental health and physical health

We had two outcome variables, the extent to which COVID-19 negatively affected a participant’s mental health and the extent to which COVID-19 negatively affected physical health. Response categories included “not at all”, “a little”, “a moderate amount”, “a lot”, and “a great deal”. We compared those who reported “a great deal” to “all others”.

#### Self/parental immigration status

This was the primary independent variable in our study. We categorized groups as undocumented immigrant students, U.S. citizen students with at least one undocumented parent, and U.S. citizen students whose parents have lawful immigration status. Undocumented students had to identify as being born outside of the United States and having no permanent legal status (e.g. no legal status, DACA, or another liminal legal status). Those with at least one undocumented parent had to identify as being born in the U.S. and have at least one immigrant parent with no permanent legal status. Those with immigrant parents with lawful status had to identify as being born in the U.S. and having parents who were lawful permanent residents or U.S. citizens, either naturalized or U.S. born.

#### Campus belonging

The survey included four items that asked about sense of belonging to the university, seeing self as part of the university community, being enthusiastic about the university, and being able to present the whole authentic self on campus. Each item had five responses, ranging from strongly disagree to strongly agree. We summed across all four items to get a single score, which we mean-centered; a higher score indicated greater feelings of campus belonging.

#### Campus resources

Campus resource use was created by summing nine items measuring the frequency that respondents visited general campus resources broadly offered to the student body, such as academic support services, basic needs/food pantry, immigration-related legal services, student health center, and mental health counseling center. Response categories included “never”, “a few times a year”, “about once a month”, “about once a week”, and “more than once a week”. We mean-centered the sum and a higher score indicated more utilization of campus resources.

#### Covariates

Covariates in our models included controls for race/ethnicity (Latina/o/x or not Latina/ox), gender (women or men), year in school (1st and 2nd years, 3rd years, and 4th years or higher), GPA (under 2.5 and 2.5 and over), campus, mother’s education level (less than high school, high school diploma/GED or some college, and bachelor’s degree or higher), and self and family economic strain (responses of yes or no to three questions: ever helped family members pay the bills, expect family will experience bad times such as poor housing or not having enough to eat, and expect family will have to do without the basic things your family needs).

### Analysis

We ran univariate descriptive statistics and bivariate statistics comparing COVID-related mental health, physical health, campus belonging, and campus resource use across the three student groups. Next, we conducted a series of logistic regression models to test our hypotheses. The first model examined the odds of reporting mental or physical health being affected “a great deal” by COVID by our immigration groups, adjusting for all of our covariates. This model provides adjusted differences in our outcomes across our three student groups of interest. The second model included campus belonging, interactions between campus belonging and student group, and all covariates. Because campus belonging is mean-centered, the coefficients for the student group differences can be interpreted as the odds for COVID-related mental or physical health outcomes at the mean campus belonging score. The interaction terms can be interpreted as the differences in the relationship between campus belonging and COVID outcomes relative to the referent group. The third model included campus resource use, interactions between campus resource use and student group, and all covariates. Similarly, because campus resource use is mean-centered, the coefficients for the student group differences can be interpreted as the odds for COVID-related mental or physical health outcomes at the mean campus resource use score. Likewise, the interaction terms can be interpreted as the differences in the relationship between campus resource use and COVID outcomes relative to the referent group. We calculated and graphed predicted probabilities for significant interactive effects to aid interpretation. We performed the same set of regression models for each of our outcome variables. All analyses were conducted using Stata 16.

## Results

### Descriptive

Table 1 provides the descriptive statistics of our sample. Within our sample, 49.2% were U.S. citizens whose parents have lawful immigration status, while 24.6 and 26.1% were U.S. citizens with undocumented immigrant parents and undocumented students, respectively. The majority self-identified as Latina/o/x race/ethnicity (69.2%) and female gender (76.6%). The highest proportion of students were 1st and 2nd year students (43.1%) followed by 4th years and higher (30.0%) and 3rd years (27.0%).

**Table 1:** Descriptive Statistics of UC Students Impacted by COVID-19, 2020 UCPromISE Data (*n* = 2112)

	Number	Percent
Student Group		
Undocumented students	552	26.1
U.S. citizens with undocumented parents	520	24.6
U.S. citizens with lawfully present immigrant parents	1039	49.2
Latino/a/x Race/Ethnicity		
No	651	30.8
Yes	1460	69.2
Year in School		
1st and 2nd Years	909	43.1
3rd Year	569	27.0
4th Years and Higher	633	30.0
Gender		
Female/Women	1618	76.6
Male/Man	493	23.4
Mother’s Education Level (or primary guardian)		
Less than HS	1022	48.4
HS/GED or Some College	760	36.0
Bachelors Degree or Higher	329	15.6
Campus		
UC Berkeley	145	6.9
UC Davis	197	9.3
UC Irvine	365	17.3
UC Los Angeles	244	11.6
UC Merced	213	10.1
UC Riverside	347	16.4
UC Santa Barbara	219	10.4
UC Santa Cruz	250	11.8
UC San Diego	131	6.2
GPA		
Under 2.5	86	4.1
Over 2.5	2025	95.9
Food Security Status		
High or marginal food security	1005	47.6
Low food security	445	21.1
Very low food security	661	31.3
Help family members pay the bills		
No	856	40.5
Yes	1255	59.5
Expect family will experience bad times such as poor housing or having not enough food		
No	1008	47.7
Yes	1103	52.3
Expect your family will have to do without the basic things that your family needs		
No	1090	51.6
Yes	1021	48.4

Table 2 compares differences by student group. The majority of participants reported that their mental health and physical health were negatively affected by COVID-19. There were significant differences in mental health; 29.0% of undocumented students reported being affected “a great deal” compared to 22.5% of citizens with undocumented parents, and 21.2% of citizens whose parents have lawful status (χ2 = 12.6, *p* = 0.002). The differences in physical health by student group were not significant, as 21.0% of undocumented students reported being affected “a great deal” compared to 19.2% of citizens with undocumented parents, and 16.9% of citizens whose parents have lawful status. While feelings of campus belonging were significantly different over the three categories, the percentages were similar across groups (F = 3.2, df = 2, *p* < .05). There were significant differences in campus resource use. Undocumented students used the most resources, followed by citizens with undocumented parents, and citizens whose parents have lawful status used the least (F = 66.30, df = 2, *p* < .05).

**Table 2:** Group Differences of UC Students, 2020 UCPromISE Data (n = 2112)

	Mean (Standard Deviation) or Percent (%)			
	Undocumented students (***n*** = 552)	U.S. citizens with undocumented parents (***n*** = 520)	U.S. citizens with lawfully present immigrant parents (***n*** = 1039)	Total Sample (***n*** = 2111)
Extent COVID-19 negatively affected your mental health^a^				
All others	71.0	77.5	78.8	76.5
A great deal	29.0	22.5	21.2	23.5
Extent COVID-19 negatively affected your physical health				
All others	79.0	80.8	83.1	81.4
A great deal	21.0	19.2	16.9	18.6
Campus Belonging (range 4–20)^b^	14.4 (3.2)	13.8 (3.4)	14.4 (3.5)	14.3 (3.4)
Use of Campus Resources (range 0–36)^b^	7.9 (5.2)	6.7 (4.9)	5.2 (3.9)	6.3 (4.7)

^a^*p* < .05 chi-square test

^b^*p* < .05 F-test (ANOVA)

### Regression results

#### Mental health

Table 3 shows the results of the regressions for negative mental health impact. Citizen students with undocumented parents had significantly lower odds for mental health impact compared to their peers with lawfully present immigrant parents (Model 1; OR = 0.66, 0.48–0.89 95%CI) while undocumented students had no difference. After adding an interaction term between group and campus belonging (Model 2), the difference in mental health impact between citizens with undocumented parents and citizens with lawfully present immigrant parents remained nearly identical (Model 2: OR = 0.66, 0.48–0.89 95%CI) at the mean of campus belonging. Higher campus belonging score was associated with lower odds of mental health being affected a “great deal” due to COVID among the students with lawfully present immigrant parents (Model 2; OR = 0.95, 0.91–0.99 95% CI). There were no significant interactions between group and campus belonging.

**Table 3:** Logistic Regression Results Immigration Status x Campus Belonging or Campus Resources on COVID-related mental or physical health effects, 2020 UCPromISE Data (n = 2112)

	Model 1				Model 2				Model 3			
	OR	95% CI		p	OR	95% CI		p	OR	95% CI		p
**Mental Health**												
Group												
U.S. citizens with lawfully present parents	referent	–	–		referent	–	–		referent	–	–	
Undocumented students	0.95	0.72	1.26	0.73	0.96	0.72	1.28	0.78	0.94	0.70	1.26	0.67
U.S. citizens with undocumented parents	0.66	0.48	0.89	0.01	0.66	0.48	0.89	0.01	0.62	0.45	0.85	< 0.001
Campus Belonging					0.95	0.91	0.99	0.02				
Group * Campus Belonging												
U.S. citizens with lawfully present parents * Belonging					referent	–	–					
Undocumented Students* Belonging					1.00	0.93	1.08	0.97				
U.S. citizens with undocumented parents* Belonging					1.04	0.96	1.12	0.32				
Campus Resource Use									1.05	1.01	1.09	0.02
Group * Campus Resource Use												
U.S. citizens with lawfully present parents * Resource									referent	–	–	
Undocumented Students* Resource									0.96	0.91	1.01	0.14
U.S. citizens with undocumented parents * Resource									1.01	0.95	1.06	0.85
**Physical Health**												
Group												
U.S. citizens with lawfully present parents	referent	–	–		referent	–	–		referent	–	–	
Undocumented students	0.82	0.60	1.11	0.20	0.83	0.61	1.13	0.24	0.77	0.56	1.06	0.11
U.S. citizens with undocumented parents	0.73	0.53	1.01	0.05	0.69	0.50	0.96	0.03	0.70	0.50	0.97	0.03
Campus Belonging					0.94	0.89	0.98	0.01				
Group * Campus Belonging												
U.S. citizens with lawfully present parents * Belonging					referent	–	–					
Undocumented Students* Belonging					1.08	0.99	1.17	0.08				
U.S. citizens with undocumented parents* Belonging					0.97	0.89	1.05	0.40				
Campus Resource Use									1.05	1.01	1.09	0.03
Group * Campus Resource Use												
U.S. citizens with lawfully present parents* Resource									referent	–	–	
Undocumented Students* Resource									0.99	0.93	1.04	0.60
U.S. citizens with undocumented parents* Resource									1.00	0.94	1.06	0.95

All models controlled for Latina/a/x ethnicity, gender, year in school, current GPA, campus, mother’s education, and self and family economic strain

The results for campus resources were very similar. Citizen students with undocumented parents continued to show an advantage in COVID-related mental health vis-à-vis students with lawfully present immigrant parents (Model 3: OR = 0.62; 0.45–0.85 95%CI) at the mean of campus resource use. Higher campus resource use was associated with a higher odds of mental health being affected “a great deal” due to COVID among the students with lawfully present immigrant parents (Model 3; OR = 1.05, 1.01–1.09 95%CI). The interaction terms were not significant.

#### Physical health

**Fig. 1:**
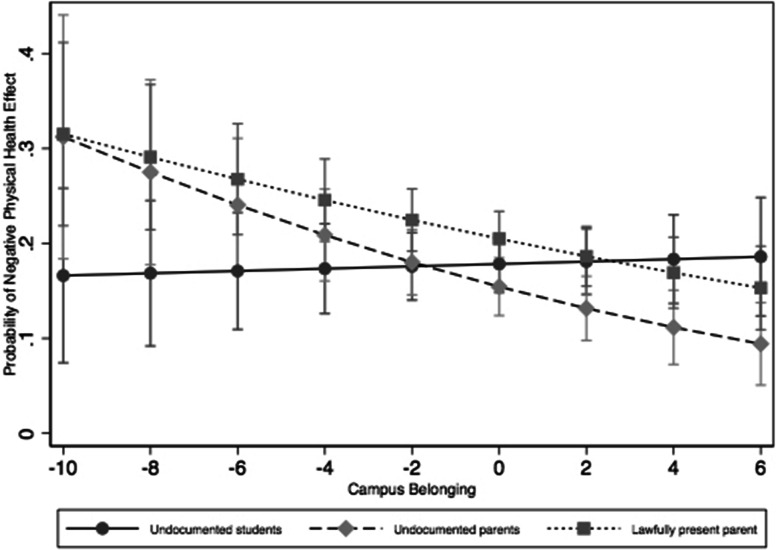
Predicted Probability of Negative Physical Health Effects by Immigration Status and Campus Belonging

Citizens with undocumented parents had marginally lower odds of reporting their physical health being affected “a great deal” by COVID compared to citizens with lawfully present immigrant parents (Model 1; OR = 0.73, 0.53–1.01 95% CI). After adding an interaction term between group and campus belonging, the difference between citizens with undocumented parents and those with lawfully present immigrant parents grew and was significant (Model 2: OR = 0.69, 0.50–0.96 95%CI) at the mean of campus belonging. Higher campus belonging score was associated with lower odds of physical health being affected a “great deal” due to COVID among the students with lawfully present immigrant parents (Model 2; OR = 0.94, 0.89–0.98 95%CI). There was a marginally significant interaction between undocumented students and campus belonging relative to the students with lawfully present immigrant parents (Model 2; OR = 1.08, 0.99–1.17 95%CI). Figure 1 graphs the predicted probabilities from Model 2. Undocumented students did not display any relationship between campus belonging and COVID-related physical health. The slope of the line between campus belonging and poor COVID-related physical health outcomes was not significantly different from zero for the undocumented students but was significantly different from zero for citizens with undocumented parents (β-.014; *p* < .05) and with lawfully present immigrant parents (β-.01; *p* < .05).

For campus resource use, citizens with undocumented parents showed an advantage in COVID-related physical health vis-à-vis their peers with lawfully present immigrant parents (Model 3: OR = 0.70; 0.50–0.97 95%CI) at the mean of campus resource use. Campus resource use was associated with a higher odds of physical health being affected “a great deal” due to COVID among the students with citizen parents (Model 3; OR = 1.05, 1.01–1.09 95% CI). The interaction terms were not significant, meaning that the same pattern holds for the other groups as well.

### Sensitivity analyses

We conducted our analyses only on Latina/o/x-identified students (supplemental table). The patterns were qualitatively similar, but the results for campus belonging did not reach significance, nor did the interaction terms for campus belonging and immigration status.

## Discussion

This paper tested differences in COVID-related mental and physical health by personal and parental immigration status. We expected undocumented students to report more severe mental and physical health effects due to COVID, but they did not differ from citizen students with lawfully present immigrant parents. Citizen students with undocumented parents actually were less likely to report “a great deal” of COVID-related mental and physical health effects than their peers with lawfully present immigrant parents, which was contrary our hypothesis. The reasons for this are unclear, but one possibility may be related to pre-existing stressors among the undocumented students and students with undocumented parents. As we discussed in the introduction, these students are already dealing with stressors in higher education related to their and their families’ liminal legal statuses. Stress inoculation theory suggests that undocumented students and students with undocumented parents may develop resistance to pervasive influences [29]. Qualitative work has found that undocumented students normalize chronic immigration-related stressors, which prohibits them from recognizing or acknowledging their mental health needs [30]. While the COVID pandemic was stressful, it may not have drastically increased their overall stress levels. In other words, the appearance of fewer or similar COVID-related mental and physical health effects relative to students with lawfully present immigrant parents may have been a result of the latter group faring poorly in the face of a serious, acute stressor such as the COVID pandemic.

One might also hypothesize that undocumented students anticipated support, minimizing the perceived effects of COVID on their mental and physical health. This would make sense given the UC system’s pre-existing commitment to providing extensive academic, financial, and socio-emotional support services to undocumented students, and many campuses’ eventually offering institutionally funded COVID emergency grants to undocumented students. California also offers an inclusive policy context, including some access to healthcare and commitments to limit deportation threats [31]. However, qualitative responses from undocumented students in this study suggest that this was likely not the case, given their high perceptions of exclusion from covid-related relief at all levels [32]. Further, the apparent mental and physical health advantage that citizen students with undocumented parents displayed could have been the bolstered by federal COVID-related support. Though families with undocumented members were not eligible for stimulus checks, citizen college students were eligible for CARES Act emergency grants.

We additionally considered whether campus psychosocial and instrumental resources exacerbated the negative impacts of COVID. If students reported using campus resources more frequently before the pandemic, the likelihood that their mental and physical health was affected “a great deal” because of the pandemic rose. This relationship suggests that separation from campus-based resources was detrimental to college students’ mental and physical health during the early stages of the pandemic. Alternatively, students who used these resources most often could have been the most vulnerable to the threats to mental health and well-being from the pandemic. This was true for all groups, regardless of personal or parental immigration status. This finding underscores the importance of these resources and campuses could target high-resource need students while they operate at limited capacity.

We found the opposite to be true for campus belonging: students who reported higher levels of campus belonging had a lower likelihood of reporting negative physical and mental effects from COVID. Past research has shown that having a sense of belonging influences physical and mental health through the encouragement of health-promoting behaviors and a belief that one’s needs will be met by larger social networks [33, 34]. Further, UC undocumented students have been found to have high levels of belonging due to their relatively high inclusion at the state and institutional level [35]. It’s possible that students who were well-integrated before the pandemic were hardier overall and better able to withstand pandemic-related strains. These students could have also been virtually maintaining relationships that contributed to their high feelings of belonging after campus closures, which could have buffered any pandemic-related strains. The one exception to these patterns was for undocumented students, who did not seem to experience the same protective effects of pre-COVID campus belonging in physical health. In qualitative work, undocumented students view their immigration status as one of many identities by which they make social connections and feel a sense of belonging on campus [36]. For these students in our study, perhaps the sources of campus belonging varied widely enough to produce null results in physical health.

Our study had some limitations. This was a cross-sectional study, so we cannot ascertain the direction of proposed relationships between immigration status, campus resources, and COVID-related mental and physical health. The study was also collected during the pandemic, so it is possible that contemporaneous conditions affected students’ pre-pandemic perceptions of their campus environment. The timing of the survey may also have affected students’ negative mental and physical effects. The survey was conducted when shut-downs were starting and there was a great deal of uncertainty about the virus transmission, future instructional plans in higher education, and economic stability. Our results may be reflecting the confusion and abrupt upheaval early in the pandemic. We also used self-reported measures in this survey and response bias, recall bias, and social desirability bias may exist. For instance, there are more women in our undocumented student sample than there are in the larger undocumented student population. We attempt to control for these response biases by including covariates in our models. We acknowledge that our results may also be subject to response bias, as we cannot verify that students were answering these questions accurately. However, the prevalence of mental conditions (depression, anxiety) asked elsewhere in the survey match levels found in other in other national surveys of college students that used the same measures and were fielded during the same time [3]. While we do not use these measures in this paper, the comparable levels provide some assurance that the students are assessing their COVID-related mental health accurately. Finally, we acknowledge that our sample population is from the University of California, which might represent a unique population of students in the state. This is a more selective system in the state (relative to the California State University system) and has more traditional college students, which may have minimized differences by the three students groups. Furthermore, the institutional support for undocumented students in the UC [37] may have contributed to the better mental and physical health outcomes among this group. Replicating this study among students in less supportive institutions may produce different results. Future research with larger individual campus sizes should consider differences in these relationships by campus as well.

These limitations are countered by several strengths. This is the first large-scale survey we are aware of that collects detailed personal and parental information and COVID-related outcomes in this population. Our study offered a unique opportunity to examine the pandemic’s early toll on the trajectories of first-generation, low-income, and racial/ethnic minority college students. Our results underscore the importance of the campus environment in students’ mental and physical health and the possibility of their role in buffering the negative health impacts of the pandemic.

## Supplementary Information

**Additional file 1.** Survey Instrument for the UC Collaborative to Promote Immigrant and Student Equity Online Survey, 2020.

**Additional file 2.** Supplemental Table 1. Logistic Regression Results Immigration Status x Campus Belonging or Campus Resources on COVID-related mental or physical health effects, 2020 UCPromISE Data, Latinos only.

## Data Availability

The datasets used and/or analyzed during the current study are available from the corresponding authors on reasonable request.

## Abbreviations

COVID: Coronavirus disease of 2019
CARES Act: Coronavirus aid, relief, and economic security act
DACA: Deferred action for childhood arrivals
GED: General Education Development (High school equivalency exam)
GPA: Grade point average
UC: University of California
UC PROMISE: University of California Collaborative to Promote Immigrant and Student Equity

## References

[1] Lederer AM, Hoban MT, Lipson SK, Zhou S, Eisenberg D. More Than Inconvenienced: The Unique Needs of U.S. College Students During the COVID-19 Pandemic. Health Educ Behav. 2020:1090198120969372. 10.1177/1090198120969372. 10.1177/1090198120969372 PMC8356799 33131325

[2] Wang X, Hegde S, Son C, Keller B, Smith A, Sasangohar F (2020). Investigating mental health of US college students during the COVID-19 pandemic: cross-sectional survey study. J Med Internet Res.

[3] Healthy Minds Network, American College Health Association. The Impact of COVID-19 on College Student Well-Being. Ann Arbor; 2020.

[4] Son C, Hegde S, Smith A, Wang X, Sasangohar F (2020). Effects of COVID-19 on college students’ mental health in the United States: interview survey study. J Med Internet Res.

[5] Patel V. Covid-19 Is a Pivotal Moment for Struggling Students. Can Colleges Step Up? Chronicle of Higher Education; 2020.

[6] Ochoa E, Ochoa G. Love, empathy, and universal grades (opinion). Latino Rebels. 2020; https://www.latinorebels.com/2020/04/08/loveempathyuniversalgrades/. Accessed 8 Jan 2020.

[7] Feldblum M, Hubbard S, Lim A, Penichet-Paul C, Siegel H. Undocumented Students in Higher Education: How Many Students Are in U.S. Colleges and Universities, and Who Are They? Washington, DC; 2020.

[8] Passel JS, Krogstad JM, Barrera-Gonzales A. As Growth Stalls, Unauthorized Immigrant Population Becomes More Settled. Pew Res Cent Hisp Trends. 2014.

[9] Hayes J, Hill L. Undocumented immigrants in California. San Francisco; 2017.

[10] Artiga S, Rae M. Health and financial risks for noncitizen immigrants due to the COVID-19 pandemic. San Francisco; 2020. https://www.kff.org/report-section/health-and-financial-risks-for-noncitizen-immigrants-due-to-the-covid-19-pandemic-issue-brief/. Accessed 1 Aug 2020

[11] Parker K, Horowitz JM, Brown A. About half of lower-income americans report household job or wage loss due to covid-19. Pew Res Center. 2020.

[12] Poston B, Barboza T, Reyes-Velarde A. Younger blacks and Latinos are dying of Covid-19 at higher rates in California: The Los Angeles Times; 2020.

[13] Anguiano V. Undocumented students generated up to $132 million in relief to colleges—but they Won’t receive a dime from the stimulus: Center for American Progress; 2020. https://www.americanprogress.org/issues/education-postsecondary/news/2020/05/05/484505/undocumented-students-generated-132-million-relief-colleges-wont-receive-dime-stimulus/. Accessed 10 Oct 2020.

[14] Bean F, Brown S, Bachmeier JD (2013). Parents without papers: the Progress and pitfalls of Mexican American integration.

[15] Yoshikawa H (2012). Immigrants raising citizens: undocumented parents and their children.

[16] Enriquez L (2020). Of love and papers: how immigration policy affects romance and family.

[17] McGorry PD, Purcell R, Goldstone S, Amminger GP. Age of onset and timing of treatment for mental and substance use disorders: implications for preventive intervention strategies and models of care. Curr Opin Psychiatry. 2011;24 https://journals.lww.com/co-psychiatry/Fulltext/2011/07000/Age_of_onset_and_timing_of_treatment_for_mental.8.aspx. 10.1097/YCO.0b013e3283477a09 21532481

[18] Terriquez V (2015). Dreams delayed: barriers to degree completion among undocumented community college students. J Ethn Migr Stud.

[19] Rodriguez C (2019). Latino/a citizen children of undocumented parents negotiating illegality. J Marriage Fam.

[20] Enriquez LE, Millán D. Situational triggers and protective locations: conceptualising the salience of deportability in everyday life. J Ethn Migr Stud. 2021;47(9):2089-108. 10.1080/1369183X.2019.1694877.

[21] Sudhinaraset M, Ling I, To TM, Melo J, Quach T (2017). Dreams deferred: contextualizing the health and psychosocial needs of undocumented Asian and Pacific islander young adults in northern California. Soc Sci Med.

[22] Dreby J (2015). Everyday illegal: when policies undermine immigrant families.

[23] Strayhorn TL (2012). College students’ sense of belonging: a key to educational success for all students.

[24] Gopalan M, Brady ST (2019). College students’ sense of belonging: a National Perspective. Educ Res.

[25] Hausmann LRM, Schofield JW, Woods RL. Sense of belonging as a predictor of intentions to persist among African American and white first-year college students. Res High Educ. 2007.

[26] EAB. Addressing College Students’ Basic Needs. Washington, D.C.; 2018.

[27] Goldrick-Rab S, Richardson J, Kinsley P. Guide to assessing basic needs insecurity in higher education. Philadelphia; 2018.

[28] Qualtrics (2020). Qualtrics.

[29] Compton J, Jackson B, Dimmock JA (2016). Persuading others to avoid persuasion: inoculation theory and resistant health attitudes. Front Psychol.

[30] Cha BS, Enriquez LE, Ro A (2019). Beyond access: psychosocial barriers to undocumented students’ use of mental health services. Soc Sci Med.

[31] Pastor M (2018). State of resistance: what California’s dizzying descent and remarkable resurgence mean for America’s future.

[32] Enriquez LE, Rosales WE, Chavarria K, Morales Hernandez M, Valadez M. COVID on Campus: Assessing the Impact of the Pandemic on Undocumented College Students. AERA Open. 2021;7(1):1-19. 10.1177/23328584211033576.

[33] Thoits PA (2011). Mechanisms linking social ties and support to physical and mental health. J Health Soc Behav.

[34] Perry BL, Pescosolido BA (2010). Functional specificity in discussion networks: the influence of general and problem-specific networks on health outcomes. Soc Networks.

[35] Golash-Boza T, Valdez Z (2018). Nested contexts of reception: undocumented students at the University of California. Central Sociol Perspect.

[36] Valdez Z, Golash-Boza T (2020). Master status or intersectional identity? Undocumented students’ sense of belonging on a college campus. Identities..

[37] Enriquez LE, Ayon C, Chavarria K, Ellis B, Hagan M, Jefferies J, et al. Persisting Inequalities and Paths Forward: A Report on the State of Undocumented Students in California’s Public Universities. Irvine; 2020.

