# Supplementary material for: Physical and mental health impacts of the COVID-19 pandemic among college students who are undocumented or have undocumented parents
Source: BMC Public Health. 2021 Aug 21;21:1580. doi: 10.1186/s12889-021-11606-x (PMC8379579; doi:10.1186/s12889-021-11606-x)
Supplement: Supplementary file 1 — Additional file 1. Survey Instrument for the UC Collaborative to Promote Immigrant and Student Equity Online Survey, 2020. [file 12889_2021_11606_MOESM1_ESM.pdf]

UC PromISE | UC COLLABORATIVE TO PROMOTE IMMIGRANT AND STUDENT EQUITY

UC PromISE Study:

Educational Experiences and Wellbeing of University of California Undergraduate Students with Immigrant Parents

Spring 2020 Survey Instrument

Q1 Do you consent to participate in this study?

○ Yes ○ No

Send to end of survey:
If response to *Q1 Do you consent to participate in this study?* is *No*.

Q2 How old are you?

▼ under 18 ... 81 or more

Send to end of survey:
If response to *Q2 How old are you?* is *Under 18*.

Q3 Are you currently enrolled as an undergraduate college student?

○ Yes ○ No

Send to end of survey:
If response to *Q3 Are you currently enrolled as an undergraduate college student?* is *No*.

Q4 What campus are you currently enrolled at?

▼ UC Berkeley ... Other

Send to end of survey:
If response to *Q4 What campus are you currently enrolled at?* is *Other*.

Q5 Do you have at least one immigrant parent?

○ Yes ○ No

Send to end of survey:
If response to *Q5 Do you have at least one immigrant parent?* is *No*.

Q6 What country were you born in?

▼ Other (1) ... Zimbabwe (197)

Send to end of survey:
If response to *Q6 What country were you born in?* is *United States of America*.

Q6_TXT If you selected "other" above, please specify the country here.

____

Display Q7:
If response to *Q6 What country where you born in?* is NOT *United States of America.*

Q7 What is your **current** immigration status?

- ◯ No current legal status (i.e. undocumented, non-DACA, expired visa, unauthorized)
- ◯ Deferred Action for Childhood Arrivals (DACA) recipient
- ◯ Temporary Protected Status (TPS) recipient
- ◯ Permanent resident/green card holder
- ◯ U.S. citizen (including naturalized citizen)
- ◯ Other (please specify) ________________________
- ◯ Valid visa holder (e.g. F1, J1, student visa)

Send to end of survey:
If response to *Q7 What is your current immigration status?* is any of the following: *permanent resident, U.S. citizen, Valid visa holder.*

Display Q355:
If response to *Q6 What country where you born in?* is *United States of America.*

Q355 Are one or more of your parents/guardians undocumented (e.g. no current legal status, DACA, TPS, expired visa, unauthorized)?

◯ No ◯ Yes ◯ I don't know

Send to end of survey when comparison group quota met for U.S. citizens with legal immigrant parents.
If response to *Q355 What is your current immigration status?* is *No* or *I don't know.*

The first set of questions is about your personal background.

Q8 What is your gender?

- ○ Female/Woman
- ○ Male/Man
- ○ Gender queer, gender non-conforming, non-binary gender
- ○ Prefer to self-describe ____________________

Q9 What is your relationship status?

- ○ Single, never married
- ○ Dating
- ○ Living together, not married
- ○ Married/domestic partner
- ○ Separated
- ○ Divorced
- ○ Widowed
- ○ Prefer to self-describe __________

Q10 Do you have children?

- ○ Yes
- ○ No

Q11 What best describes your race or ethnicity? Check as many as apply.

- ☐ Asian
- ☐ Black, African, or African American
- ☐ Indigenous, or Native
- ☐ Latina, Latino, Latinx, or Hispanic
- ☐ Middle Eastern
- ☐ Pacific Islander
- ☐ White
- ☐ Some other race or ethnicity (please specify): ______________________

Display Q12*:*
If response to *Q6 What country where you born in?* is NOT *United States of America.*

Q12 How old were you when you first arrived to the United States?

▼ I don't know ... Over 30

Display Q13:
If response to *Q6 What country where you born in?* is NOT *United States of America.*

Q13 Do you have any of the following? Check all that apply.

- ☐ AB-540, AB-2000, SB-68 – access to in-state college tuition rates
- ☐ California DREAM Act – access to financial aid
- ☐ Federally-issued work permit
- ☐ Pending application for legal immigration status (e.g. permanent legal residence/green card, U-visa, VAWA, refugee, asylee)
- ☐ Expired visa
- ☐ None of the above

The next set of questions is about your parents or guardians.

The questions allow you to identify up to two parents or guardians who were primarily responsible for raising you. For each parent/guardian, you will be asked to identify who you have in mind (e.g. mother, father, etc) and then you'll be asked questions about that parent/guardian specifically.

Q14 Think about one of the primary people who raised you. What is **Parent/Guardian 1's** relationship to you?

- ○ Mother (biological or adoptive)
- ○ Father (biological or adoptive)
- ○ Stepmother
- ○ Stepfather
- ○ Other (please specify) ____________

Q15 What country was Parent/Guardian 1 born in?

▼ Other (1) ... Zimbabwe (197)

Q15_TXT If you selected "other" above, please specify the country here.

____

Q16 Is Parent/Guardian 1 a U.S. citizen, permanent resident, or have some other immigration status?

- ◯ No current legal status (i.e. undocumented, non-DACA, expired visa, unauthorized)
- ◯ Deferred Action for Childhood Arrivals (DACA) recipient
- ◯ Temporary Protected Status (TPS) recipient
- ◯ Permanent resident/green card holder
- ◯ U.S. citizen (including those with dual citizenship)
- ◯ Does not live in the U.S.
- ◯ Deceased
- ◯ I don't know
- ◯ Other (please specify) ______________________

Q17 What category best describes the highest level of education Parent/Guardian 1 has completed?

- ◯ None
- ◯ 6th grade or lower
- ◯ 7th to 8th grade
- ◯ 9th to 12th grade
- ◯ High school diploma or GED
- ◯ Some college (including vocational or technical program)
- ◯ Bachelor's degree
- ◯ Post-graduate degree (e.g. M.A., Ph.D., professional degree)
- ◯ I don't know
- ◯ Other (please specify): ______________________

Q18 What category best describes Parent/Guardian 1's employment?

- ◯ Employee who works for wages or salary
- ◯ Self-employed
- ◯ Temporary/seasonal worker (e.g. seasonal agriculture, day labor)
- ◯ Unemployed and looking for work
- ◯ Not working (e.g. retired, stay-at-home parent, disabled)
- ◯ I don't know
- ◯ Other (please specify): ______________________

Q19 Do you have another parent, guardian, or primary caregiver who raised you?

- Yes
- No

Skip To: Q25
If response to *Q19 Do you have another parent, guardian, or primary caregiver who raised you?* is *No.*

Q20 What is **Parent/Guardian 2's** relationship to you?

- Mother (biological or adoptive)
- Father (biological or adoptive)
- Stepmother
- Stepfather
- Other (please specify) __________

Q21 What country was Parent/Guardian 2 born in?

▼ Other (1) ... Zimbabwe (197)

Q21_TXT If you selected "other" above, please specify the country here.

____

Q22 Is Parent/Guardian 2 a U.S. citizen, permanent resident, or have some other immigration status?

- No current legal status (i.e. undocumented, non-DACA, expired visa, unauthorized)
- Deferred Action for Childhood Arrivals (DACA) recipient
- Temporary Protected Status (TPS) recipient
- Permanent resident/green card holder
- U.S. citizen (including those with dual citizenship)
- Does not live in the U.S.
- Deceased
- I don't know
- Other (please specify) ________________________

Q23 What category best describes the highest level of education Parent/Guardian 2 has completed?

- ◯ None
- ◯ 6th grade or lower
- ◯ 7th to 8th grade
- ◯ 9th to 12th grade
- ◯ High school diploma or GED
- ◯ Some college (including vocational or technical program)
- ◯ Bachelor's degree
- ◯ Post-graduate degree (e.g. M.A., Ph.D., professional degree)
- ◯ I don't know
- ◯ Other (please specify): ________________________

Q24 What category best describes Parent/Guardian 2’s employment?

- ◯ Employee who works for wages or salary
- ◯ Self-employed
- ◯ Temporary/seasonal worker (e.g. seasonal agriculture, day labor)
- ◯ Unemployed and looking for work
- ◯ Not working (e.g. retired, stay-at-home parent, disabled)
- ◯ I don't know
- ◯ Other (please specify): ________________________

Q25 Please select the **current** immigration status of the other following groups of people.

If you have multiple people in a group who have different statuses, please check multiple boxes. For example, if you have one sibling who has DACA and one who is a U.S. citizen, check both boxes.

	Not Applicable (N/A)	No current legal status (undocumented, non-DACA, expired visa)	Deferred Action for Childhood Arrivals (DACA)	Temporary Protected Status (TPS)	Permanent resident/green card holder	U.S. citizen	Other immigration status	Does not live in the U.S.	I don't know
Sibling(s)									
Current romantic partner [1]									
Children [2]									
Extended family member(s) who **live in your permanent household** (e.g. grandparents, aunts, uncles, cousins, in-laws, etc)									
Extended family member(s) who **do not live** in your permanent household									

[1] Display this item:
If response to *Q9 What is your relationship status?* is NOT *Single, never married.*
[2] Display this item:
If response to *Q10 Do you have children?* is *Yes.*

Q25_TXT If you would like to provide any clarifying information on the previous question, please do so here.

____

Q26 How often do you complete the following family responsibilities?

	Almost never or never	Once in a while	Sometimes	A lot of the time	Almost always or always
Help family members pay the bills					
Support family members with childcare, elder care, transportation, etc					
Help manage the health of a family member					
Do household chores					
Provide emotional support to your family members					

Q27 Thinking about your family's current economic situation, indicate how often you expect that your family will face the following circumstances **in the next <u>three</u> months**.

	Almost never or never	Once in a while	Sometimes	A lot of the time	Almost always or always
Your family will experience bad times such as poor housing or not having enough food					
Your family will have to do without the basic things that your family needs					

The next set of questions is about your household and living situation.

Q28 Some students have multiple residences. The following question is about the place you consider your **permanent home**. This does <u>not</u> refer to temporary housing such as dorms, or short-term leases for housing near campus.

How many people, including yourself, currently live in your **permanent home**?

▼ 1 (1) ... I don't know (99)

Q29 What was your estimated total **household** income in 2019?

*This includes everyone who lives in your **permanent home**, including yourself, and helps pay for shared expenses.*

▼ $0 (0) ... I don't know (99)

The following questions are about the place you live when school is in session. Please answer these questions based on your living situation before the COVID-19/coronavirus crisis occurred.

Q30 What best characterizes your current living situation while school is in session?

- ○ Living at home with family
- ○ On-campus housing
- ○ Off-campus housing, not with family
- ○ Other (please specify): __________

Q31 On a typical school day, how long does it take you to get to campus?

▼ Less than 15 minutes (1) ... I don’t know (99)

The next set of questions is about detention and deportation.

These questions may be triggering. You can skip these questions and proceed to the next section if desired.

Q32 Please rate how frequently you think about the following people's deportation.

	Never	A few times a year	About once a month	About once a week	Daily
Your own deportation					
Your parent(s)/guardian(s) deportation					
Your extended family members' deportation					
Your friends' deportation					

Q33 Have you or any of your family or friends been in deportation proceedings, detained, or deported?

- ○ Yes
- ○ No
- ○ I don't know
- ○ Decline to state

Skip To: Q35
If response to *Q33 Have you or any of your family or friends been in deportation proceedings, detained, or deported?* is NOT *Yes.*

Q34 Please check off which of the following people have been involved with detention/deportation procedures since entering the United States.

If multiple people in the same category have experienced different procedures, check multiple boxes.

For example, if you have a parent who is currently in deportation proceedings and another who was deported, check both of those boxes.

If the same person had multiple types of interactions, check the most recent type of procedure.

For example, if you had a sibling who was held in a detention center and then was deported, check "was deported" because that happened most recently.

	Not Applicable (N/A)	In deportation proceedings	Held in a detention center	Was deported	Other type of detention/deportation procedure
Yourself					
Parent(s)/guardian(s)					
Sibling(s)					
Romantic partner(s)					
Children [1]					
Extended family member(s) who live in your permanent household (e.g. grandparents, aunts, uncles, cousins, in-laws, etc)					
Extended family member(s) who do not live in your permanent household					
Friend(s)					

[1] Display this item:
If response to *Q10 Do you have children?* is *Yes.*

Q34_TXT If you would like to provide any clarifying information on the previous question, please do so here.

____

The next set of questions is about your personal finances and work. We define work broadly to include formal and informal employment as well as participating in an internship or fellowship program for which you receive a stipend or scholarship.

If the COVID-19/coronavirus crisis changed your work situation, please answer these questions based on your work situation before the crisis occurred.

Q35 Are you currently working for some type of financial compensation?

- ○ Yes
- ○ No
- ○ I don't know
- ○ Decline to state

Skip To: Q38
If response to *Q35 Are you currently working for some type of financial compensation?* is *No.*

Q36 About how many hours do you spend working in a typical week (7 days)?
If you work multiple jobs, include all hours worked among all jobs.

- ○ 0 hours
- ○ 1 to 5 hours
- ○ 6 to 10 hours
- ○ 11 to 15 hours
- ○ 16 to 20 hours
- ○ 21 to 25 hours
- ○ 26 to 30 hours
- ○ 31 to 35 hours
- ○ 36 to 40 hours
- ○ 41 to 45 hours
- ○ 46 to 50 hours
- ○ More than 50 hours
- ○ Other
- ○ I don’t know

Q37 What is your hourly pay rate?
If you work multiple jobs, provide the rate for the job where you work the most hours.

____

Q38 Please rate the extent to which you agree or disagree with the following statements.

	Strongly disagree	Disagree	Neither agree nor disagree	Agree	Strongly agree
I worry that I will have to take time off from school to save money to pay for school.					
I am concerned that I will not be able to finance my college education.					

Q39 Indicate how often you have experienced the following since starting school this year.

	Almost never or never	Once in a while	Sometimes	A lot of the time	Almost always or always
Worried about not having enough money to pay for things					
Had difficulty paying your bills					
Had to go without the basic things that you need					
Had to go without the materials needed for your studies (e.g. books, laptop, iclicker, art/lab supplies)					

Q40 These statements are about general feelings you have about yourself in relation to others. Please indicate the extent to which you agree or disagree with each statement.

	Strongly disagree	Disagree	Disagree somewhat	Agree somewhat	Agree	Strongly agree
Deep down, I believe that I am as worthy as others.						
I deserve to be treated the same as other people are treated.						
I am as worthy as others of getting my needs met.						
Everyone is worthy of having their needs met.						
I feel as though I matter in the world.						
I matter to the people in my community (however you define your community).						
Thinking about the country as a whole, I don't matter as much as everyone else.						
Please choose “strongly agree” for this question.						
I deserve to be respected in my community.						
I think I'm worthy of equal access to societal resources (e.g. healthcare, education, public safety).						

The next set of questions is about current immigration policy.

Q41 How frequently do you check for updates about immigration policy?

- ○ Never
- ○ Rarely
- ○ Sometimes
- ○ Often

Q42_1 In general, do you think that the **United States government** tries to make immigrants feel:

- Very unwelcome
- Somewhat unwelcome
- Somewhat welcome
- Very welcome

Q42_2 In general, do you think that the **California government** tries to make immigrants feel:

- Very unwelcome
- Somewhat unwelcome
- Somewhat welcome
- Very welcome

Q42_3 In general, do you think that **your local government** tries to make immigrants feel:

- Very unwelcome
- Somewhat unwelcome
- Somewhat welcome
- Very welcome

The next set of questions are about your experiences and feelings about current immigration policy. Please indicate how frequently you have felt the following way in your day to day interactions.

These questions may be triggering. You can skip these questions and proceed to the next section if desired.

Q43_1 Do you feel that you have no liberty and need to stay home because of current immigration policy?

- Never
- Rarely
- Sometimes
- Often
- Always

Q43_2 Have you been treated unfairly at a restaurant or store because of current immigration policy?

- Never
- Rarely
- Sometimes
- Often
- Always

Q43_3 Have you been silenced by others or felt that your opinion did not matter because of current immigration policy?

- Never
- Rarely
- Sometimes
- Often
- Always

Q43_4 Do others act like they have the right to treat you unfairly or poorly because of current immigration policy?

- Never
- Rarely
- Sometimes
- Often
- Always

Q43_5 Do you avoid certain locations like parks and neighborhoods because you do not feel safe given current immigration policy?

- Never
- Rarely
- Sometimes
- Often
- Always

Q43_6 Do you fear being deported or detained?

- Never
- Rarely
- Sometimes
- Often
- Always

Q43_7 Have you ever been treated like a criminal because of current immigration policy?

- Never
- Rarely
- Sometimes
- Often
- Always

Q43_8 Were you treated poorly because of your English language skills and/or accent given current immigration policy?

- Never
- Rarely
- Sometimes
- Often
- Always

Q43_9 Do other members of your ethnic group treat you poorly because of current immigration policy?

- Never
- Rarely
- Sometimes
- Often
- Always

Q43_10 Do you feel ignored when seeking help because of current immigration policy?

- Never
- Rarely
- Sometimes
- Often
- Always

Q43_11 Do you feel unsafe when leaving your home because of current immigration policy?

- Never
- Rarely
- Sometimes
- Often
- Always

Q43_12 Do you feel that that you have no rights because of current immigration policy?

- Never
- Rarely
- Sometimes
- Often
- Always

Q43_13 Have you been humiliated because of who you are given current immigration policy?

- Never
- Rarely
- Sometimes
- Often
- Always

Q43_14 Do you feel that you have been exploited or taken advantage of at work because of current immigration policy?

- Never
- Rarely
- Sometimes
- Often
- Always

Q43_15 Do you worry about the impact immigration policies have on you or your family?

- Never
- Rarely
- Sometimes
- Often
- Always

Q43_16 Do you fear that you or a family member will be reported to immigration officials?

- Never
- Rarely
- Sometimes
- Often
- Always

Q43_17 Do you worry about family separation due to deportation?

- Never
- Rarely
- Sometimes
- Often
- Always

Q44 There are many ways to try to deal with problems. These items deal with ways you've been **coping with the stress in your life related to current immigration policies.**

We want to know to what extent you've been doing what the item says. How much or how frequently. Don't answer on the basis of whether it seems to be working or not - just whether or not you're doing it.

We recognize that the COVID-19/coronavirus crisis has also created stress that you have to cope with. Do not think about coping with this type of stress when answering these questions.

	I haven't been doing this at all	I've been doing this a little bit	I've been doing this a medium amount	I've been doing this a lot
I've been turning to work or other activities to take my mind off things				
I've been concentrating my efforts on doing something about the situation I'm in.				
I've been saying to myself "this isn't real."				
I've been using alcohol or other drugs to make myself feel better.				
I've been getting emotional support from others.				
I've been giving up trying to deal with it.				
I've been taking action to try to make the situation better.				
I've been refusing to believe that it has happened.				
I've been saying things to let my unpleasant feelings escape.				
I've been getting help and advice from other people.				
I've been using alcohol or other drugs to help me get through it.				
I've been trying to see it in a different light, to make it seem more positive.				
I've been criticizing myself.				
I've been trying to come up with a strategy about what to do.				
I've been getting comfort and understanding from someone.				
I've been giving up the attempt to cope.				
I've been looking for something good in what is happening.				
I've been making jokes about it.				
I've been doing something to think about it less, such as going to the movies, watching TV, reading, daydreaming, sleeping, or shopping.				
Please choose "I've been doing this a lot" for this question.				
I've been accepting the reality of the fact that it has happened.				
I've been expressing my negative feelings.				

I've been trying to find comfort in my religion or spiritual beliefs.

I've been trying to get advice or help from other people about what to do.

I've been learning to live with it.

I've been thinking hard about what steps to take.

I've been blaming myself for things that happened.

I've been praying or meditating.

I've been making fun of the situation.

The next set of questions is about your conversations with others about the current immigration policy context.

Q45 Please indicate how often you engage in the following actions.

	Never	Rarely	Sometimes	Often	Almost all the time
Talked with others about *immigration or immigration policy*.					
Talked with others about the *challenges faced by immigrants*.					
Spoken up when you have heard people make *false statements about immigrants* (e.g., immigrants are lazy and live off welfare).					
Encouraged *others to speak up* when they hear people make false statements about immigrants.					
Talked with someone about overhearing others make demeaning comments about immigrants.					
Confronted someone because you heard them use the word “illegal” or other derogatory terms for immigrants.					
Wanted to change the subject when you have heard people talking about immigrants in a demeaning way.					
Talked with others about what immigration policy *means for immigrants*.					

The next set of questions is about your political engagement.

Display Q46:
If response to *Q6 What country where you born in?* is *United States of America.*

Q46 We know that most people don't vote in all elections. Usually between one-quarter to one-half of those eligible actually come out to vote. How often do you vote in local and national elections?

- Never
- Rarely
- Sometimes
- Often
- Always
- I don't know

Q47 Do you talk to people and try to show them why they should vote for or against one of the parties or candidates?

- Never
- Rarely
- Sometimes
- Often
- Always
- I don't know

Q48 Below is a list of things that some people do to express their views. For each one, identify how often you do it.

	Never	Rarely	Sometimes	Often	I don't know
Contact a public official - at any level of government - to express your opinion					
Take part in a protest, march, or demonstration, or rally **on-campus**					
Take part in a protest, march, or demonstration, or rally **off-campus**					
Sign a petition regarding an issue or problem that concerns you					
Boycott a company or product for social or political reasons					
Buy a certain product or service because you like the social or political values of the company					
Discuss political issues on social media					
Wear buttons or display stickers with social or political messages					
Expressed a political point of view during a class discussion					

The second portion of the survey focuses on mental health and wellbeing.

This next set of questions is about your use of mental health services.

All of your answers are confidential.

Q49 Have you **ever sought help** of any kind for your mental or emotional health?

- ○ Yes
- ○ No
- ○ I don't know
- ○ Decline to state

Skip To: Q52
If response to *Q49 Have you ever sought help of any kind for your mental or emotional health?* is NOT *Yes.*

Q50 Who have you sought help from for your mental or emotional health? Check as many as apply.

- ☐ On-campus professional therapist
- ☐ Off-campus professional therapist
- ☐ Professor
- ☐ Professional staff member
- ☐ Friend
- ☐ Family member
- ☐ Religious or spiritual leader
- ☐ Other (please specify) ____________
- ☐ Decline to state

Display Q51:
If response to *Q50 Who have you sought help from for your mental or emotional health?* included the selection of one or more of the following items: *On-campus professional therapist, Off-campus professional therapist, Other.*

Q51 Have you ever participated in therapy for three or more sessions?

- ○ Yes
- ○ No
- ○ Decline to state

Q52 Was there ever a time during **this academic year** when you **felt that you might need to see a professional** because of problems with your mental health, emotions, or nerves?

- ○ Yes
- ○ No
- ○ I don't know
- ○ Decline to state

Q53 The next set of questions ask you about your feelings and thoughts during the **last 7 days**. Please indicate how often you felt or thought a certain way.

Please answer this question with regard to what was typical, before the COVID-19/coronavirus crisis occurred.

	Never	Almost never	Sometimes	Often	Almost Always
Been upset because of something that happened unexpectedly					
Felt that you were unable to control the important things in your life					
Felt nervous and "stressed"					
Felt confident about being able to handle your personal problems					
Felt that things were going your way					
Found that you could not cope with all the things that you had to do					
Been able to control irritations in your life					
Felt that you were on top of things					
Been angered because of things that happened that were outside of your control					
Felt that your difficulties were piling so high that you could not overcome them					

The next set of questions is about your health.

Q54 Would you say that in general your health is excellent, very good, good, fair, or poor?

- ○ Poor
- ○ Fair
- ○ Good
- ○ Very good
- ○ Excellent

Q55 What is your **primary** source of health insurance?

- ◯ I have a university Student Health Insurance Plan
- ◯ I have MediCal
- ◯ I am covered by my parent/guardian's plan
- ◯ I am covered by my employer-based plan (or my spouse/partner's employer-based plan)
- ◯ I bought a plan on my own
- ◯ I have health insurance, but I do not know the primary source
- ◯ I do not have health insurance
- ◯ I do not know if I have health insurance
- ◯ Other (please specify) ____

The next set of questions is about your psychological state.

Please answer these questions with regard to what was <u>typical</u>, before the COVID-19/coronavirus crisis occurred.

These questions may be triggering. You can skip these questions and proceed to the next section if desired.

Q56 Over the **last two weeks**, how often have you been bothered by any of the following problems?

	Not at all	Several days	More than half the days	Nearly every day
Little interest or pleasure in doing things				
Feeling down, depressed, or hopeless				
Trouble falling or staying asleep, or sleeping too much				
Feeling tired or having little energy				
Poor appetite or overeating				
Feeling bad about yourself - or that you're a failure or have let yourself or your family down				
Trouble concentrating on things, such as reading the newspaper or watching television				
Moving or speaking so slowly that other people could have noticed. Or the opposite - being so fidgety or restless that you have been moving around a lot more than usual				
Thoughts that you would be better off dead, or hurting yourself in some way				

Q57 Over the **last two weeks**, how often have you been bothered by the following problems?

	Not at all	Several days	More than half the days	Nearly every day
Feeling nervous, anxious, or on edge				
Not being able to stop or control worrying				
Worrying too much about different things				
Trouble relaxing				
Being so restless that it is hard to sit still				
Becoming easily annoyed or irritable				
Feeling afraid, as if something awful might happen				

The next set of questions is about the food you have eaten since the beginning of this academic year.

Q58 Please identify whether the following statements are often true, sometimes true, or never true since the beginning of this academic year.

	Never true	Sometimes true	Often true	I don't know
The food I bought just didn't last, and I didn't have money to get more.				
I couldn't afford to eat balanced meals.				

Q58_3 Since the beginning of this academic year, did you ever **cut the size of your meals** or **skip meals** because there wasn't enough money for food?

- Yes, almost every month
- Yes, some months but not every month
- Yes, only 1 or 2 months
- No
- I don't know

Q58_4 Since the beginning of this academic year, did you ever **eat less** than you felt you should because there wasn't enough money for food?

- Yes
- No
- I don't know

Q58_5 Since the beginning of this academic year, were you ever **hungry but didn't eat** because there wasn't enough money for food?

- Yes
- No
- I don't know

The next set of questions is about your social networks.

Q59 This scale is made up of a list of statements each of which may or may not be true about you. Please indicate your level of agreement with each statement.

	Definitely false	Probably false	Probably true	Definitely true
If I wanted to go on a trip for a day (for example to the beach, the country, or mountains), I would have a hard time finding someone to go with me.				
I feel that there is no one I can share my most private worries and fears with.				
If I were sick, I could easily find someone to help me with my daily chores.				
There is someone I can turn to for advice about handling problems with my family.				
If I decide one afternoon that I would like to go to a movie that evening, I could easily find someone to go with me.				
When I need suggestions on how to deal with a personal problem, I know someone I can turn to.				
I do not often get invited to do things with others.				
If I had to go out of town for a few weeks, it would be difficult to find someone who would look after my house or apartment (the plants, pets, garden, etc.).				
If I wanted to have lunch with someone, I could easily find someone to join me.				
If I was stranded 10 miles from home, there is someone I could call who could come and get me.				
If a family crisis arose, it would be difficult to find someone who could give me good advice about how to handle it.				
If I needed some help in moving to a new house or apartment, I would have a hard time finding someone to help me.				

The third portion of the survey focuses on your educational experiences.

The next set of questions is about your educational history.

Q60 What was your high school GPA?

▼ Less than 1.0 ... I don't know

Q61 Did you begin attending {CAMPUS} as a first-year student or a transfer student?

- ○ First-year student
- ○ Transfer student
- ○ Other (please specify): __________

Q62 What year are you?

- First year undergraduate (e.g. freshman)
- Second year undergraduate (e.g. sophomore)
- Third year undergraduate (e.g. junior)
- Fourth year undergraduate (e.g. senior)
- Fifth year or more undergraduate
- Other (please specify) ________________________
- I don't know

Q63 In what area is your primary major?

- Art
- Biological and life sciences
- Business
- Education
- Engineering
- Medicine and health professions
- Humanities
- Interdisciplinary
- Math and computer science
- Physical science
- Public and social service
- Social science
- Undecided/undeclared
- Other (please specify): ___________

Q64 Since you started at {CAMPUS}, have you taken any quarters/semesters off (excluding summer or other intersessions)?

- No, never
- Yes, 1 quarter/semester
- Yes, more than 1 quarter/semester
- I don't know

The next set of questions is about your academic performance.

Q65 What is your **overall** GPA at {CAMPUS}?

- 0.00-0.24
- 0.25-0.49
- 0.50-0.74
- 0.75-0.99
- 1.00-1.24
- 1.25-1.49
- 1.50-1.74
- 1.75-1.99
- 2.00-2.24
- 2.25-2.49
- 2.50-2.74
- 2.75-2.99
- 3.00-3.24
- 3.25-3.49
- 3.50-3.74
- 3.75-3.99
- 4.0
- I don't know

Q66 Have you ever failed a course at {CAMPUS}?

- Yes
- No
- I don't know

Q67 Have you ever earned a place on the Dean's List or Honor Roll at {CAMPUS}?

- Yes
- No
- I don't know

Q68 How many units are you currently enrolled in?

Most classes are 3-4 units. Students must be enrolled in 12 units to be considered full-time and eligible for financial aid.

▼ 0 ... I don't know

The next set of questions is about immigration policy's impact on your educational experiences.

Display Q69:
If response to *Q6 What country where you born in?* is NOT *United States of America.*

Q69 How frequently have the following occurred during **this academic year** because you were dealing with or thinking about an issue related to **your** **immigration status**?

	Never	A few times a year	About once a month	About once a week	Daily
Missed class					
Was distracted in class					
Lost needed study hours					
Did poorly on an exam					

Q70 How frequently have the following occurred during **this academic year** because you were dealing with or thinking about an issue related to **your family members' immigration status**?

	Never	A few times a year	About once a month	About once a week	Daily
Missed class					
Was distracted in class					
Lost needed study hours					
Did poorly on an exam					

Q71 How frequently during **this academic year** have you done each of the following?

Please answer this question with regard to what was typical, before the COVID-19/coronavirus crisis occurred.

	Never	Rarely	Sometimes	Often	N/A (Not Applicable)
Failed to turn in a course assignment					
Gone to class unprepared					
Skipped class					
Sought academic help from instructor or tutor when needed					
Studied with a group of classmates outside of class					
Contributed to a class discussion					
Please choose "often" for this question.					
Communicated with the instructor outside of class about issues and concepts derived from a course					

Q72 Please rate the extent to which you agree or disagree with the following statements.

	Strongly disagree	Disagree	Neither agree nor disagree	Agree	Strongly agree
Thinking about life after graduation gives me anxiety.					
I feel prepared to achieve my career goals.					
I worry about whether I will be able to use my degree after graduation.					

The next set of questions is about your extracurricular experiences.

Display Q73:
If response to *Q6 What country where you born in?* is NOT *United States of America.*

Q73 Please rate the extent to which you agree or disagree with the following statements.

	Strongly disagree	Disagree	Neither agree nor disagree	Agree	Strongly agree
My immigration status has prevented me from taking advantage of career preparation opportunities.					
I can pursue my desired career regardless of my immigration status.					

Q74 Below are various opportunities that college students may take part in. Check all that you have participated in while at {CAMPUS}?

- ☐ Unpaid internship
- ☐ Paid internship
- ☐ Credit-based internship, practicum, or field experience
- ☐ Member of an **on-campus** organization
- ☐ Member of an **off-campus** organization
- ☐ Held a career-relevant job
- ☐ Research experience (not as a research subject)
- ☐ Graduate school preparation program
- ☐ Study abroad
- ☐ Campus exchange program (such as UCDC)
- ☐ Other (please specify): ________________________
- ☐ None of the above

Q75 Have you ever participated in **any organization** that tried to **solve a problem** at your school, in the community, or in the broader society?

- ○ Yes, I have done it this academic year
- ○ Yes, I have done it in the past but not this academic year
- ○ No, I have not done it
- ○ I don't know

Display Q76:
If response to *Q75 Have you ever participated in any organization ...* is one of the following: *Yes, I have done it this academic year,* or *Yes, I have done it in the past but not this academic year.*

Q76 Have you ever been you in a **leadership position in an organization that tried to solve a** problem at your school, in the community, or in the broader society while enrolled at {CAMPUS}?

- ○ Yes, I have done it this academic year
- ○ Yes, I have done it in the past but not this academic year
- ○ No, I have not done it
- ○ I don't know

Display Q77:
If response to *Q75 Have you ever participated in any organization ...* is *Yes, I have done it this academic year.*

Q77 How often do you **participate in organizational activities** that try to **solve a problem** at your school, the community, or broader society?

- ○ Never
- ○ Rarely
- ○ Sometimes
- ○ Often

Q78 Have you ever spent time participating in any community service or volunteer activity?

A volunteer activity means working in some way to help others for no pay.

- ○ Yes, I have done it this academic year
- ○ Yes, I have done it in the past but not this academic year
- ○ No, I have not done it
- ○ I don't know

The next set of questions is about how you feel on and around your university campus.

Q79 Please rate the extent to which you agree or disagree with the following statements.

	Strongly disagree	Disagree	Neither agree nor disagree	Agree	Strongly agree
I feel a sense of belonging to this university.					
I see myself as part of the university community.					
I am enthusiastic about this university.					
I can present my whole, authentic self on campus without worrying about repercussions.					

Q80 How often have you heard or witnessed the following groups express **positive feelings** about undocumented immigrant communities?

	Never	Rarely	Sometimes	Often
Faculty				
Staff				
Students				
Members of the community surrounding {CAMPUS}				

Q81 How often have you heard or witnessed the following groups express **negative feelings** about undocumented immigrant communities?

	Never	Rarely	Sometimes	Often
Faculty				
Staff				
Students				
Members of the community surrounding {CAMPUS}				

Display Q82:
If response to *Q6 What country where you born in?* is NOT *United States of America.*

Q82 During **this academic year**, have you experienced the following:

	No, never	Yes, 1-3 times	Yes, more than 3 times
Been denied access to a campus resource or program because of your immigration status			
Had to educate a university staff person about your eligibility to receive a service or resource			
Been given inaccurate or incorrect information about how to complete a university procedure (ie. enrollment, filling a form) because the staff person didn't know the correct procedures for someone with your immigration status			

Display Q83:
If response to *Q6 What country where you born in?* is NOT *United States of America.*

Q83 Please rate the extent to which you agree or disagree with the following statements

	Strongly disagree	Disagree	Neither agree nor disagree	Agree	Strongly agree
It takes a lot of time to get an answer about something related to being an undocumented student.					
It is stressful to get an answer about something related to being an undocumented student.					
My immigration status prevents me from accessing the resources I need on campus.					

The next set of questions is about campus resources.

Q84 Please identify how frequently you have visited the following offices or services at {CAMPUS} during **this academic year**?

Please answer this question with regard to what was typical, before the COVID-19/coronavirus crisis occurred.

	Never	A few times a year	About once a month	About once a week	More than once a week	I don't think this exists on my campus
Academic counselor						
Academic support services (e.g. writing center, EOP, tutoring center)						
Peer tutoring						
Career center						
Identity-based center (e.g. women’s center, LGBTQ center; multicultural center)						
Basic needs/food pantry						
Immigration-related legal services						
Student health center						
Mental health counseling center						

Q91 Given the changes due to COVID-19/coronavirus, are you using campus services more, less, or about the same?

- ◯ A lot less
- ◯ Somewhat less
- ◯ About the same
- ◯ Somewhat more
- ◯ A lot more

Q85 Have you ever been to an office or met with a staff person at {CAMPUS} who focuses on supporting undocumented students and/or students with undocumented family members?

- ◯ Yes
- ◯ No
- ◯ I don't know
- ◯ Decline to state

Display this introduction:
If response to *Q85 Have you ever been to an office or met with a staff person at ...* is *Yes, I don't know,* or *Decline to state.*

The following questions ask about your interactions with offices or staff at {CAMPUS} who focus on supporting undocumented students and/or students with undocumented family members. These offices have different names, such as Dream Centers or Undocumented Student Programs.

Please answer this question with regard to what was typical, before the COVID-19/coronavirus crisis occurred.

Display Q86:
If response to *Q85 Have you ever been to an office or met with a staff person at ...* is *Yes, I don't know,* or *Decline to state.*

Q86 Please identify how frequently you have done the following **this academic year**?

	Never	A few times a year	About once a month	About once a week	More than once a week	I don't think this exists on my campus
Visited the undocumented student program office/center						
Spoke with a professional staff member from the undocumented student program office/center						
Spoke with a student staff member from the undocumented student program office/center						
Spoke with a campus partner (e.g. academic counselor, mental health counselor) at the undocumented student program office/center						
Spoke with an immigration lawyer or legal service provider who was provided by your campus or university system						

Display Q87:
If response to *Q85 Have you ever been to an office or met with a staff person at ...* is *Yes, I don't know,* or *Decline to state.*

Q87 Undocumented student programs often organize their own events aimed at supporting undocumented communities. Have you attended any of the following types of events offered by undocumented student programs **this academic year**?

	No, 0 events	Yes, 1 event	Yes, more than 1 event	I don't think this exists on my campus
Academic event				
Finance-related event (e.g. CA Dream Act workshop, scholarship session, financial wellness workshop)				
Immigration law-related event (e.g. Know Your Rights workshop, DACA renewal clinic; do not include individual legal advising sessions)				
Professional development event				
Social event				
Wellness event				
Another type of event				

Display Q88:
If response to *Q85 Have you ever been to an office or met with a staff person at ...* is *Yes, I don't know,* or *Decline to state.*

Q88 Have undocumented student program staff connected you to another person on campus who could provide support, services, or resources?

- ○ No, never
- ○ Yes, 1 other person
- ○ Yes, more than 1 other person
- ○ I don't know

Display Q89
If response to *Q85 Have you ever been to an office or met with a staff person at ...* is *No.*
OR
If responses to ALL five items in *Q86 Please identify how frequently you have done the following this academic year?* are *Never*

Q89 {CAMPUS} has an office and at least one professional staff member who focuses on supporting undocumented students and/or students with undocumented family members. These offices have different names, such as Dream Centers or Undocumented Student Programs. To what extent do the following reasons explain why you have not visited this office?

	Not at all	Somewhat	A lot
I did not know it exists.			
Other offices on campus can provide me with the resources and opportunities I need.			
These resources are not for students like me.			
I do not feel comfortable in the office.			

Q90 Please rate the extent to which you agree or disagree with the following statements.

	Strongly disagree	Disagree	Slightly disagree	Neither agree nor disagree	Slightly agree	Agree	Strongly agree
I lead a purposeful and meaningful life							
My social relationships are supportive and rewarding							
I am engaged and interested in my daily activities							
I actively contribute to the happiness and well-being of others							
I am competent and capable in the activities that are important to me							
I am a good person and live a good life							
I am optimistic about my future							
People respect me							

These final questions are about the COVID-19/coronavirus pandemic and how it has affected you.

Q92 To what extent have the following areas been negatively affected by the COVID-19/coronavirus pandemic?

	Not at all	A little	A moderate amount	A lot	A great deal
Your financial stability					
Your family's financial stability					
Your mental health					
Your physical health					
Your academic performance (e.g. grades)					
Your attention to your academics					

Q93 Please describe how the COVID-19/coronavirus pandemic has affected you and your family.

____
____
____
____
____

Q94 Has your or your family member's immigrant origin affected your experience of the COVID-10/coronavirus pandemic? If so, please describe how.

____
____
____
____
____
