# Supplementary material for: Physical and mental health impacts of the COVID-19 pandemic among college students who are undocumented or have undocumented parents
Source: BMC Public Health. 2021 Aug 21;21:1580. doi: 10.1186/s12889-021-11606-x (PMC8379579; doi:10.1186/s12889-021-11606-x)
Supplement: Supplementary file 2 — Additional file 2. Supplemental Table 1. Logistic Regression Results Immigration Status x Campus Belonging or Campus Resources on COVID-related mental or physical health effects, 2020 UCPromISE Data, Latinos only. [file 12889_2021_11606_MOESM2_ESM.docx]

Supplemental Table 1. Logistic Regression Results Immigration Status x Campus Belonging or Campus Resources on COVID-related mental or physical health effects, 2020 UCPromISE Data, **Latinos only**

|  | **Model 1** | | | |  | **Model 2** | | | |  | **Model 3** | | | |
| --- | --- | --- | --- | --- | --- | --- | --- | --- | --- | --- | --- | --- | --- | --- |
|  | **OR** | **95% CI** | | **p** |  | **OR** | **95% CI** | | **p** |  | **OR** | **95% CI** | | **p** |
| **Mental Health** |  |  |  |  |  |  |  |  |  |  |  |  |  |  |
| Group |  |  |  |  |  |  |  |  |  |  |  |  |  |  |
| U.S. citizens with lawfully present parents | referent | - | - |  |  | referent | - | - |  |  | referent | - | - |  |
| Undocumented students | 1.00 | 0.73 | 1.37 |  |  | 1.02 | 0.74 | 1.39 |  |  | 0.98 | 0.70 | 1.36 |  |
| U.S. citizens with undocumented parents | 0.69 | 0.50 | 0.95 | * |  | 0.69 | 0.50 | 0.95 | * |  | 0.65 | 0.47 | 0.90 | * |
|  |  |  |  |  |  |  |  |  |  |  |  |  |  |  |
| Campus Belonging |  |  |  |  |  | 0.97 | 0.91 | 1.03 |  |  |  |  |  |  |
| Group * Campus Belonging |  |  |  |  |  |  |  |  |  |  |  |  |  |  |
| U.S. citizens with lawfully present parents * Belonging |  |  |  |  |  | referent | - | - |  |  |  |  |  |  |
| Undocumented students * Belonging |  |  |  |  |  | 0.99 | 0.91 | 1.08 |  |  |  |  |  |  |
| U.S. citizens with undocumented parents* Belonging |  |  |  |  |  | 1.03 | 0.95 | 1.13 |  |  |  |  |  |  |
|  |  |  |  |  |  |  |  |  |  |  |  |  |  |  |
| Campus Resource Use |  |  |  |  |  |  |  |  |  |  | 1.05 | 1.00 | 1.11 | * |
| Group * Campus Resource Use |  |  |  |  |  |  |  |  |  |  |  |  |  |  |
| U.S. citizens with lawfully present parent* Resource |  |  |  |  |  |  |  |  |  |  | referent | - | - |  |
| Undocumented students * Resource |  |  |  |  |  |  |  |  |  |  | 0.96 | 0.90 | 1.02 |  |
| U.S. citizens with undocumented parents* Resource |  |  |  |  |  |  |  |  |  |  | 1.00 | 0.94 | 1.07 |  |
| **Physical Health** |  |  |  |  |  |  |  |  |  |  |  |  |  |  |
| Group |  |  |  |  |  |  |  |  |  |  |  |  |  |  |
| U.S. citizens with lawfully present parents | referent | - | - |  |  | referent | - | - |  |  | referent | - | - |  |
| Undocumented students | 0.88 | 0.62 | 1.24 |  |  | 0.89 | 0.63 | 1.25 |  |  | 0.81 | 0.56 | 1.16 |  |
| U.S. citizens with undocumented parents | 0.76 | 0.54 | 1.07 |  |  | 0.72 | 0.51 | 1.02 | * |  | 0.73 | 0.51 | 1.03 | + |
|  |  |  |  |  |  |  |  |  |  |  |  |  |  |  |
| Campus Belonging |  |  |  |  |  | 0.96 | 0.90 | 1.03 |  |  |  |  |  |  |
| Group * Campus Belonging |  |  |  |  |  |  |  |  |  |  |  |  |  |  |
| U.S. citizens with lawfully present parents * Belonging |  |  |  |  |  | referent | - | - |  |  |  |  |  |  |
| Undocumented students * Belonging |  |  |  |  |  | 1.05 | 0.96 | 1.15 |  |  |  |  |  |  |
| U.S. citizens with undocumented parents* Belonging |  |  |  |  |  | 0.94 | 0.85 | 1.03 |  |  |  |  |  |  |
|  |  |  |  |  |  |  |  |  |  |  |  |  |  |  |
|  |  |  |  |  |  |  |  |  |  |  |  |  |  |  |
| Campus Resource Use |  |  |  |  |  |  |  |  |  |  | 1.03 | 0.98 | 1.09 |  |
| Group * Campus Resource Use |  |  |  |  |  |  |  |  |  |  |  |  |  |  |
| U.S. citizens with lawfully present parent* Resource |  |  |  |  |  |  |  |  |  |  | referent | - | - |  |
| Undocumented students * Resource |  |  |  |  |  |  |  |  |  |  | 1.00 | 0.94 | 1.07 |  |
| U.S. citizens with undocumented parents* Resource |  |  |  |  |  |  |  |  |  |  | 1.01 | 0.94 | 1.08 |  |

All models controlled for gender, year in school, current GPA, campus, mother’s education, and self and family economic strain
